# Stiehopus japonieus acidic mucopolysaccharide inhibits the proliferation of pancreatic cancer SW1990 cells through Hippo-YAP pathway

**DOI:** 10.18632/oncotarget.14633

**Published:** 2017-01-13

**Authors:** Xiaoyu Li, Yi Liu, Cuiping Zhang, Qinghui Niu, Hui Wang, Cong Che, Man Xie, Bin Zhou, Yonghong Xu, Qi Zhang, Jun Wu, Zibin Tian

**Affiliations:** ^1^ Department of Gastroenterology, The Affiliated Hospital of Qingdao University, Qingdao 266003, China; ^2^ Department of Gastroenterology, Shanxian Central Hospital, Heze 274000, China; ^3^ Department of Infectious Diseases, The Affiliated Hospital of Qingdao University, Qingdao 266003, China; ^4^ Department of Hepatobiliary Surgery, The Affiliated Hospital of Qingdao University, Qingdao 266003, China

**Keywords:** Hippo-YAP pathway, PDAC, SJAMP, cell proliferation

## Abstract

Previous studies have indicated that stiehopus japonieus acidic mucopolysaccharide (SJAMP) could inhibit the proliferation of pancreatic cancer cell SW1990. However, the mechanism remains unclear. In our study, YAP expression was identified by immunohistochemistry and quantitative Real-time PCR from 45 pairs of human pancreatic ductal adenocarcinoma (PDAC) tissues and their adjacent non-tumor samples. We found that the YAP expression was associated with the histological differentiation degree, and negatively correlated with pancreatic cancer patients’ survival. More YAP localization in nuclear and enhanced expression of YAP mRNA in pancreatic cancer tissue was found in comparison with in the normal tissue. These results identify YAP acts as an amazing regulator in the pathogenesis of pancreatic cancer. After affected by SJAMP, YAP and TEAD1 were down regulated, while MST1 and pYAP were upregulated gradually with the prolong of effect time. SJAMP also improved YAP phosphorylation, nuclear-to-cytoplasmic translocation and inactivation. After successfully knocked-down by YAP siRNA, the inhibition of proliferation of SJAMP to cancer cells was attenuated. Interestingly, we indicated a down-regulation of that TEAD with SJAMP 4 mg/ml, 8 mg/ml for 24 h and with 8 mg/ml SJAMP for 24 h, 48 h even after YAP silencing. That might mean that the SJAMP has other targets, not only YAP, to downregulate TEAD. We proposed a hypothesis that Hippo-YAP pathway involved in carcinogenesis of pancreatic cancer and in the inhibition effect of SJAMP to the proliferation of pancreatic cancer cell, although maybe not the sole signaling pathway.

## INTRODUCTION

Pancreatic ductal adenocarcinoma cancer (PDAC) is a kind of aggressive cancer, which 5-year survival rate less than 5% [1]. In China from 2000 to 2011, PDAC incidence and age-standardized mortality rates were increased obviously [2]. Because of diagnosis in late stage, only one third patients experienced curative-intent operation, and less than 10% of patients had chance to receive comprehensive treatment [3] and with very poor prognosis [4]. Therefore, it would be of a great clinic value to elucidate the complex mechanism on the pancreatic carcinogenesis and toward new therapeutic approaches.

Carcinogenesis results from an endless and uncontrolled proliferation and growth advantage, because of the DNA mutations and cellular malignant transformation [5–7]. In recent decades, research has characterized that several pathways highlight a pivotal role in cancer progression, including Raf-MEK-ERK signaling pathway, PI3K-PTEN-Akt-mTOR signaling pathway, and the newly emerging Hippo-YAP pathway. The ERK and Akt signaling pathways have been shown over the past 25 years to transfer signals which regulating the proliferation and apoptosis. The ERK pathway controls cell proliferation, differentiation and metastasis [8]. Phosphorylation of AKT also promotes tumorigenesis through mediating cell apoptosis and proliferation [9]. The Hippo-YAP signaling pathway plays vital roles in regulating cell differentiation, tissue regeneration, organ size, and even cancer development. YAP is a downstream target of core kinases of mammalian STE20-like protein kinase 1/2 (MST1/2) and its downstream factor, large tumor suppressor 1/2 (LATS1/2) [10]. When YAP enters the nucleus, it promotes a number of downstream gene transcriptions and exerts a pleiotropic role in tumor growth and apoptosis inhibition [11]. Overexpression of YAP was observed in differentiated cell associated with epithelial-mesenchymal transitions (EMT) and anchorage-independent proliferation [12]. Expressions of YAP enhanced in a variety of human cancers, for example, lung cancer [13], esophagus cancer [14], liver cancer [15] and ovarian cancer [16]. YAP is able to induce ERK and AKT phosphorylation [17, 18], and there are novel and complex ERK/AKT/Hippo-YAP regulatory network in oncogenesis and provides valuable indications for developing targeted therapies [19].

Stiehopus japonieus acidic mucopolysaccharide (SJAMP) is a kind of acid mucopolysaccharides extracted from stiehopus japonieus, with the properties of anti-tumor and immunomodulatory. We previous study showed that SJAMP could inhibit pancreatic cancer cells growth, promote apoptosis, and increase the percentage of G0/G1 stage [20]; however, the mechanism was unclear. According to the results of our study, we define that YAP plays a paramount role in the tumorgenesis of pancreatic cancer and SJAMP could have theraputic effects through the Hippo-YAP pathway.

## RESULTS

### YAP levels are significantly higher in pancreatic cancer tissues

**Figure 1:**
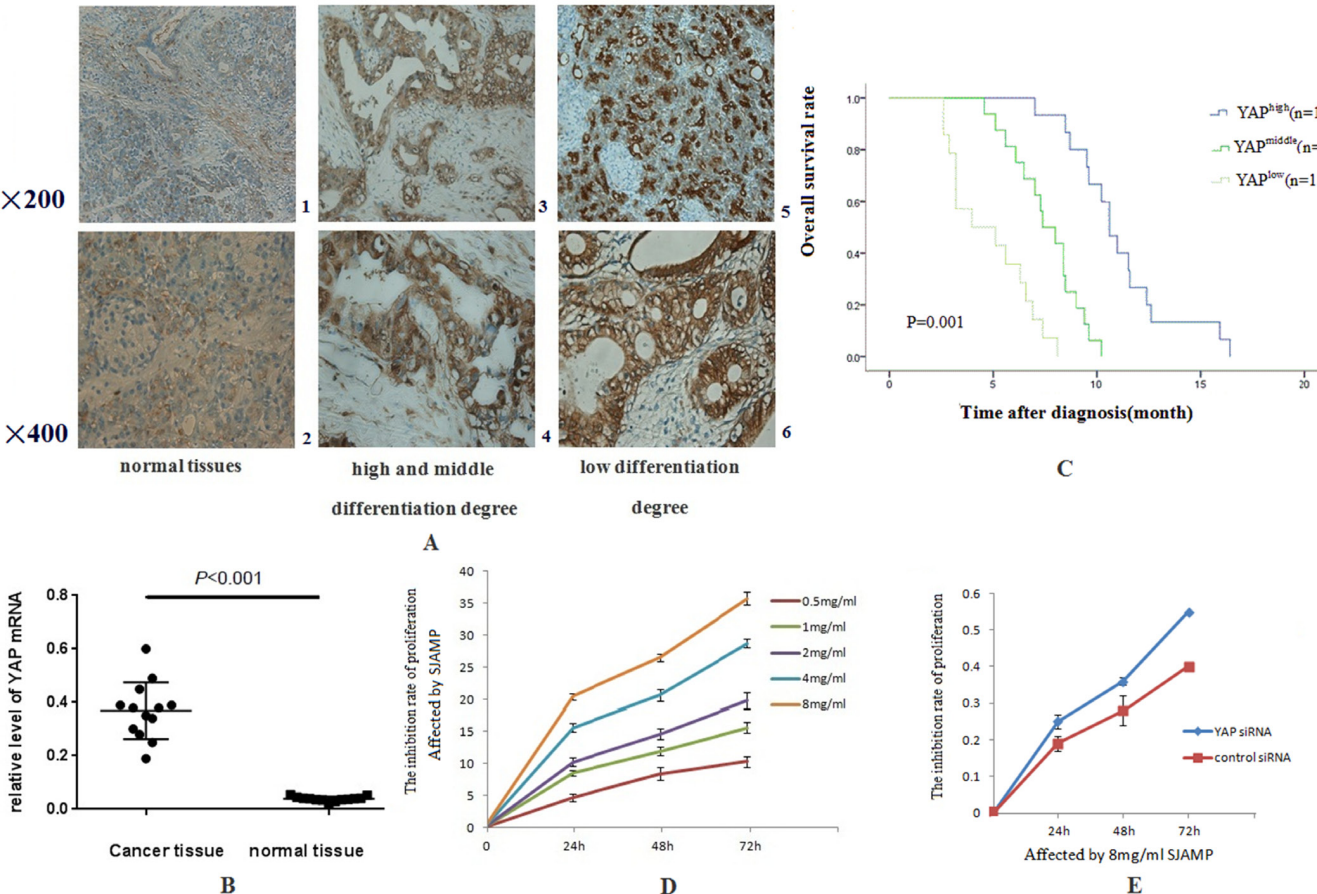
The expressions of YAP in pancreatic cancer tissues are stronger than in normal pancreatic tissues and SJAMP Inhibits the proliferation of SW1990 (**A**) 1: in normal pancreas tissues, weak, mainly located in cytoplasm (200×). 2: in normal pancreas tissues (400×). 3: in pancreatic cancer with high and middle differentiation degree, stronger than that in normal pancreatic tissue, and was located in cytoplasm and nucleus (200×). 4: in pancreatic cancer with high and middle differentiation degree (400×). 5: in pancreatic cancer with low differentiation degree was stronger than that of high and middle differentiation degree, and was located in cytoplasm and nucleus (200×). 6: in pancreatic cancer with low differentiation degree (400×). (**B**) The relative expression of YAP mRNA in PDAC elevated, which was 9.4 times compared to that in the normal tissues (0.3685 ± 0.029 vs 0.03908 ± 0.0024, *p* < 0.001). (**C**) Kaplan–Meier analysis showing that with high YAP level had shorter lifetime than those with low YAP level (*p* < 0.05). (**D**) SJAMP inhibits SW1990 cell proliferation gradually with the increase of effect dose and the prolong of effect time. (*p* < 0.05). (**E**) After successfully knocked-down by YAP siRNA, the inhibition of proliferation of SJAMP to cancer cells was attenuated (*p* < 0.05).

Previous studies have showed that YAP promoted pancreatic cancer anchorage-independent growth and cell proliferation. It was also an amazing regulator to pancreatic cancer cells motility, infiltrate and distance metastasis [21]. Our research data indicated YAP was overexpressed, located in the nucleus, and hyperactivated in pancreatic cancer tissues. Immunostaining demonstrated that YAP was low expressed in normal pancreatic ductal epithelial cell cytoplasm in adjacent non-tumor samples, partly expressed in nucleus, without expression in pancreatic acini and centroacinar cells. In pancreatic cancer tissues, YAP was intensively localized in the nuclear of the ductal epithelial cells (Figure 1A). In pancreatic cancer tissues, the positive expression rate of YAP was 93.33%, while in normal pancreatic tissues was 26.67% (*χ*^2^ = 27.95, *p* = 0.000). Elevated YAP activity/expression strongly correlated with tumor histological differentiation in pancreatic cancer tissue (*χ*^2^ = 5.294, *p* = 0.048), however, YAP has no statistical criteria with age, sex, smoking, drinking, obesity, impaired glucose tolerance, diabetes, chronic pancreatitis and clinical stage (Table 1). Collectively, all the results suggest that YAP maybe participate in the tumorigenesis of pancreatic cancer.

**Table 1:** The relationship between YAP expression in pancreatic cancer tissue strength and clinical pathological factors

	Negative	Weakly positive	Moderately positive	Strongly positive	*χ^2^*	*P*
Gender					0.643	0.577
Male	2	3	5	10		
Female	1	2	10	12		
Age					0.787	0.565
> 60	1	4	8	13		
< 60	2	1	7	9		
Smoking					1.452	0.541
Yes	3	4	9	15		
No	0	1	6	7		
Drinking					1.896	0.224
Yes	1	3	11	16		
No	2	2	4	6		
BMI					0.000	1.000
BMI > 27	2	4	9	15		
BMI < 27	1	1	6	7		
Abnormal glucose tolerance					1.773	0.542
Yes	3	3	9	14		
No	0	2	6	8		
Diabetes					0.952	0.555
Yes	1	2	8	16		
No	2	3	7	6		
Chronic pancreatitis					0.643	0.577
Yes	2	2	6	10		
No	1	3	9	12		
Differentiation					5.294	0.048*
Poorly	0	1	11	16		
Medium and high	3	4	4	6		
T stages					2.143	0.543
T1	2	1	3	9		
T2	1	2	6	6		
T3	0	2	5	6		
T4	0	0	1	1		
N stages					0.592	1.000
N0	3	3	13	19		
N1	0	2	2	3		
Clinical stages					0.153	0.926
I	2	3	11	12		
II	1	2	4	8		
III	0	0	0	2		

^*^Statistically significant

Elevated YAP activity/expression strongly correlated with tumor histological differentiation in pancreatic cancer tissue (*χ^2^* = 5.294, *p* = 0.048).

### Prognostic value of YAP in pancreatic cancer patients

Pancreatic cancer has a character of fatal malignancies and poor prognosis. We have known that the high CA199 serum level usually mean poor prognosis and recurrence of cancer [22, 23]. Our research results figured out that the relative expression of YAP mRNA in cancer tissues was 9.4 times compared to that in the normal tissues (0.3685 ± 0.029 vs 0.03908 ± 0.0024, *p* < 0.001) (Figure 1B). Importantly, patients with enhanced YAP expression had a significantly higher CA19-9 serum level compared to patients with low YAP expression (*r* = 0.652, *p* = 0.0089). We also determined the role YAP played in survival. The median overall survival (OS) was 7.0 ± 0.6 months (95% CI: 5.4–8.2 months), compared with the patients who had high expression of YAP (4.0 ± 0.6 months; 95% CI: 3.1–5.1 months; *p* = 0.015, Figure 1C) by Kaplan-Meier analysis. The univariate survival analysis indicated that low YAP expression and receives chemotherapy treatment mean longer survival time. (Table 2, *p* = 0.015 and *p* = 0.048) and had independent prognostic value in the multivariate analysis (Table 3, *p* = 0.048 and *p* = 0.033).

**Table 2:** Univariate analysis of OS

Characteristic Age (years)	Cases	Median Survival (months)	95% (CI) (months)	*P*-value
< 60	19	5.0 ± 0.6	3.6–6.5	*P* = 0.645
> = 60	26	6.0 ± 0.5	4.9–7.2	
Gender				
Male	20	5.0 ± 0.7	3.5–6.8	*P* = 0.263
Female	25	6.0 ± 0.5	5.0–7.0	
Differentiation				
Poorly	28	5.0 ± 0.7	3.5–6.5	*P* = 0.328
Medium and high	17	8.0 ± 0.5	1.8–15.2	
Treatment				
Chemical treatment	25	6.0 ± 0.8	4.0–8.2	*P* = 0.048
None	20	4.0 ± 0.4	2.7–5.6	
YAP expression				
High and moderate	37	4.0 ± 0.6	3.1–5.1	*P* = 0.015
low and none	8	7.0 ± 0.6	5.4–8.2	

Abbreviations: CI, confidence interval.

low YAP expression and chemotherapy treatment were positively correlated with the patients’ survival (*p* = 0.015 and *p* = 0.048).

**Table 3:** Multivariate analysis of OS

Variable	RR	95% CI	*P*-value
Age	0.782	0.362–1.812	0.577
Gender	0.615	0.268–1.455	0.265
Differentiation	0.579	0.178–1.917	0.382
Treatment	0.399	0.213–0.879	0.033
YAP expression	0.477	0.250–0.988	0.048

Abbreviations: CI, confidence interval; RR, relative risk.

Low YAP expression and chemotherapy treatment had independent prognostic value in the multivariate analysis. (*p* < 0.05).

### Hippo-YAP pathway involved in the inhibition of proliferation of SW1990 induced by SJAMP

**Figure 2:**
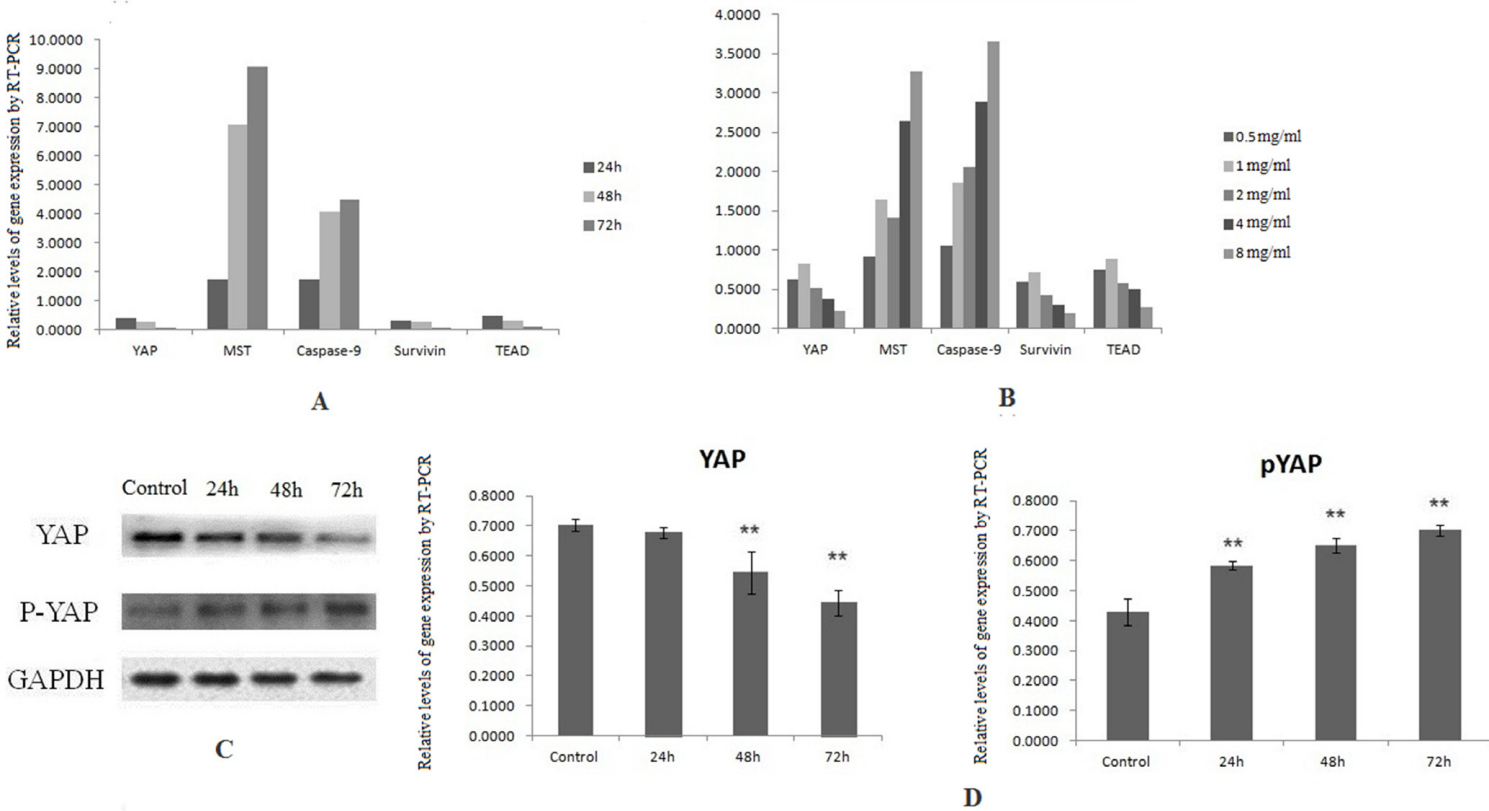
Downregulation of YAP by SJAMP contributes to inhibition pancreatic cancer cells growth (**A**) With the prolong of effect time of 8 mg/ml SJAMP on the pancreatic cancer cells, the expression level of YAP, TEAD1, survivin mRNA decreased significantly, and the expression level of Caspase-9, MST1 mRNA increased significantly. (**B**) The same tendency was observed when effected by SJAMP in different dose. (**C**) and (**D**) After affected by SJAMP, the protein level of YAP decreased and the protein level of pYAP increased obviously. *p < 0.05, **p < 0.01.

The Hippo pathway plays a paramount role in the progression as well as development of pancreatic cancer [24]. The Hippo-YAP pathway is engaged in the cell proliferation to limit organ size. In this process, Mst1/2 kinase is one of the pivotal Hippo kinase complexes, which was activated at first and then phosphorylate the other core component, as an example, Lats1/2 [25], and then the later phosphorylates YAP/TAZ. YAP has two kinds of existence forms, phosphorylation and dephosphorylation. Phosphorylation form of YAP, p-S127 YAP/14-3-3 complexes locates in the cytoplasm, binding with 14-3-3 proteins on Ser127 site [26, 27]. Dephosphorylation form of YAP translocates into the nucleus without a Hippo signal, and controls many transcription genes, interacting with members of the TEA domain-containing transcription factor family (TEAD) [7]. In our study, the results of MTT analysis showed that the proliferation of SW1990 was markedly reduced with the increase of the dose and the prolong of effect time of SJAMP. These results demonstrated that SJAMP inhibited SW1990 cell proliferation gradually with the increase of effect dose and prolong of effect time. (*p* < 0.05, Figure 1D). We also found that after successfully knocked-down by YAP siRNA, this inhibition tendency to cancer cells was attenuated (*p* < 0.05, Figure 1E). As shown in Figure 2A, we found that with the prolong of effect time of 8 mg/ml SJAMP on the pancreatic cancer cells, YAP, TEAD1, and Survivin expression significantly decreased, but the levels of the Hippo upstream gene, MST1, and Caspase-9 increased. The same tendency was observed in different dose of SJAMP (Figure 2B). Therefore, we speculated that Hippo-YAP signaling might participate in the process of SJAMP inhibiting the proliferation of pancreatic cancer cells.

### SJAMP improved the phosphorylation and nuclear-to-cytoplasmic translocation of YAP and its inactivation

**Figure 3:**
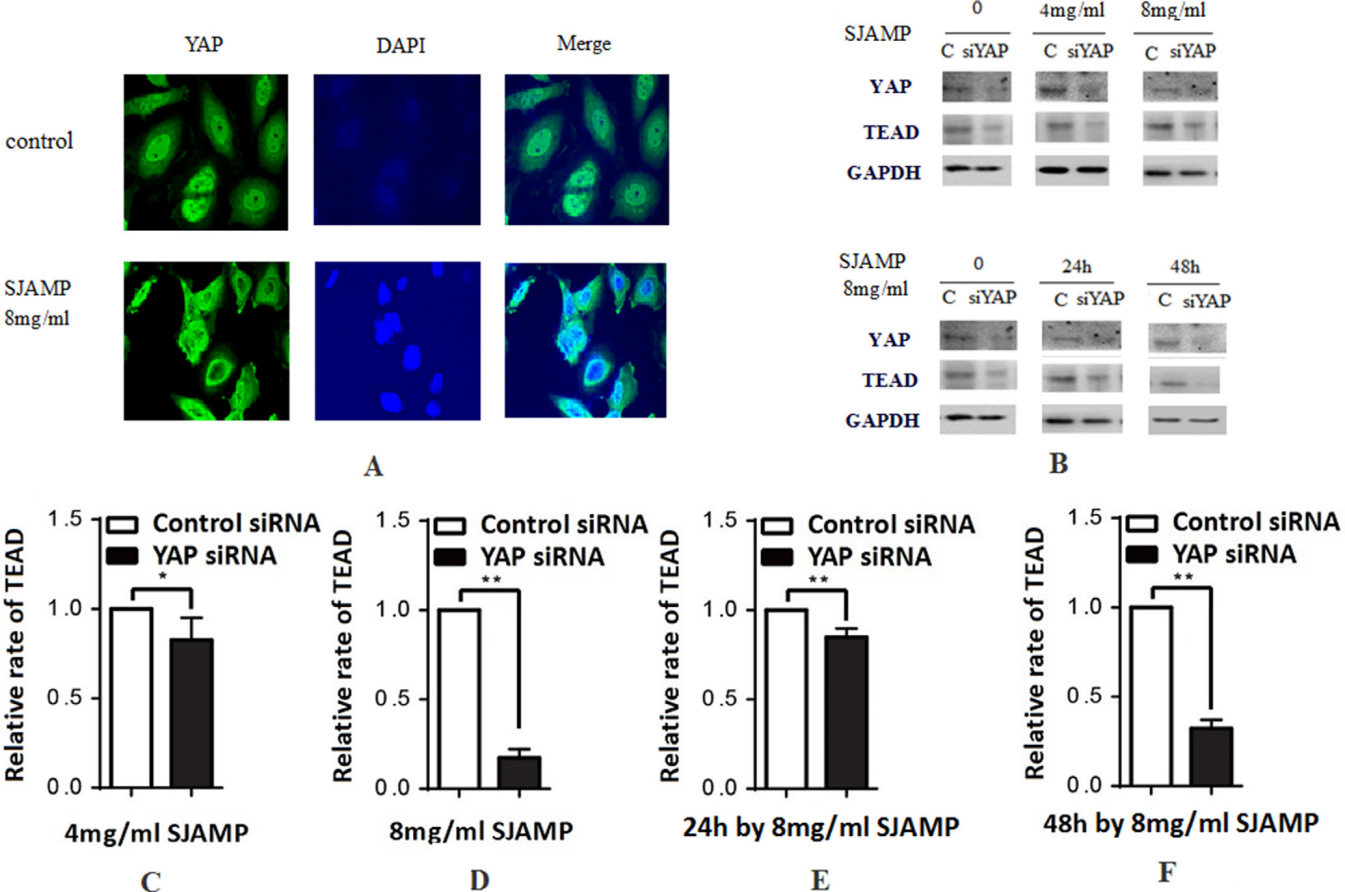
SJAMP improved the phosphorylation of YAP to inhibit the proliferation of pancreatic cancer cells (**A**) The result of immunofluorescence analysis also indicated that after effected by SJAMP, there is a nuclear-to-cytoplasmic translocation of YAP. (**B**) A downstream target gene, TEAD, is downregulated with YAP silencing. (**C**–**D**) YAP silencing also resulted in a decrease in TEAD, with SJAMP 4 mg/ml, 8 mg/ml for 24 h (*p < 0.05,**p < 0.01). (**E**–**F**) YAP silencing also resulted in a decrease in TEAD, with 8 mg/ml SJAMP for 24 h, 48 h (**p < 0.01). These results might mean that the SJAMP has other targets, not only YAP, to downregulate TEAD.

After phosphorylated, YAP localized in the cytoplasm and couldn’t shift into nuclear and lose the transcriptional activity. After transferring into the nucleus, YAP promotes the expression of the downstream transcription factor DIAP1 and Cyclin E, and has transcriptional coactivator activity toward TEAD/TEF and p73, promoting cell proliferation [27]. In our experiments, we further confirmed YAP and pYAP expression by western-blotting. After affected by SJAMP, the YAP protein expression level decreased and pYAP protein level increased significantly (Figure 2C and 2D). The result of immunofluorescence analysis also indicated that after effected by SJAMP, there is a nuclear-to-cytoplasmic translocation of YAP (Figure 3A). And then we tested one of the YAP downstream target gene, TEAD, affected by SJAMP for different dose and effect time after knocked-down YAP. Interestingly, although our previous study results indicated that SJAMP could decrease the expression of TEAD, we found that after YAP silencing there was still a decrease trend of TEAD, with SJAMP 4 mg/ml, 8 mg/ml for 24 h (*p < 0.05, **p < 0.01; Figure 3C, 3D) and with 8 mg/ml SJAMP for 24 h, 48 h (**p < 0.01, Figure 3E, 3F). That is to say that SJAMP had additional inhibition to TEAD expression even knock-down of YAP. These results might mean that the SJAMP has other targets, not only YAP, to downregulate TEAD.

## DISCUSSION

As reported here, we found that YAP was obviously increased in pancreatic cancer compared with the normal pancreatic tissues. In cancer tissues, YAP was highly located in the nuclear of pancreatic ductal epithelial cells. Increased YAP activity/expression strongly correlated with poor tumor histological differentiation. Furthermore, patients with strong positive YAP expression had a higher CA19-9 serum level compaired to patients with low YAP expression. Low YAP expression and chemotherapy treatment were positively correlated with survival and had independent prognostic value in the multivariate analysis. Our study is likely a consequence that SJAMP improved the phosphorylation and nuclear-to-cytoplasmic translocation of YAP to inhibit the cancer cell proliferation. Therefore, YAP functioned as a tumor oncogene and promoted pancreatic cancer progression. SJAMP inhibited pancreatic cancer cells growth through Hippo-YAP signal pathway and it could be a potential target for designing effective therapeutic strategies.

Studies have found in the pancreatic embryonic development, Hippo-YAP pathway participates in it and helps to maintain the pancreatic acinar cell differentiation [28]. Our data indicated that the staining intensity and extent of YAP was markedly stronger when compared with cancer tissues and normal tissues. Further analysis of the correlation of YAP activity/expression with clinicopathological parameters demonstrated that YAP expression strongly correlated with tumor histological differentiation in pancreatic cancer tissue. It showed that the YAP played an important role in the occurrence and development of pancreatic cancer. Cigarette smoking is one of the risk factors [29]. The risk in obesity and diabetes individuals also significantly elevated. New onset diabetes mellitus may be the key clues to the diagnosis in the pancreatic cancer early stage [30]. However, in our study there were no obvious differences among age, sex, smoking, drinking, obesity, impaired glucose tolerance, diabetes, chronic pancreatitis and clinical stage. Our research also indicated that YAP is negatively associated with pancreatic cancer patient survival, which suggests high probability of poor prognosis in pancreatic cancer patients. A larger number of cases than that in the present study are needed for further study.

CA19-9 is a kind of mucin glycoprotein components which is recognized to be the tumor markers of pancreatic cancer. There were some studies supported that the level of CA19-9 was negatively correlated with survival which was an important index to reflect prognosis and recurrences [31]. Our study demonstrated that serum CA19-9 levels in cancer patients had positively correlation with YAP, and the staining intensity and extent of YAP was much higher in primary cancer tissue than in normal tissue. Low YAP expression and chemotherapy treatment were positively correlated with the patients’ survival. The high YAP expression in pancreatic cancer tissue and poor differentiation degree were positively correlated to poorer prognosis and had independent prognostic value in the multivariate analysis. Thus, our results indicated that YAP may be a potential prognostic factor and may be a promising therapy target of pancreatic cancer.

SJAMP is an animal acid mucopolysaccharide extracted by the Stiehopus cell wall, which has the important roles of anticoagulant, anti-thrombosis, reducing blood fat and viscosity, antitumor, immunomodulatory, antibacterial, antiviral and promoting cell growth. Studies had shown that SJAMP inhibited cervical cancer Hela cells proliferation through inhibiting CyclinD1 and CDK4, and induce the differentiation of Hela cells by suppressing the expression of oncogene c-myc [32]. It inhibited the proliferation and inducing apoptosis of human hepatocellular carcinoma cell line HepG2, and resisted the carcinoma by changing the Bcl-2 and nm23-H1 protein expression [33].

The Hippo-YAP pathway, an evolutionarily conserved pathway, plays elementary roles in tissues growth and regeneration, as well as in cancer development [34]. MST is an important factor of Hippo pathway core kinase cascade chain, whose role is to inhibit the key components of Hippo signaling pathway to inhibit the tumor [35]. It modulates YAP phosphorylation and nuclearcytoplasmic distribution [36]. While located in the nucleus, YAP stimulates the downstream transcription factor expression to influence cell proliferation and apoptosis [37]. Our results showed that with the prolong of effected time of SJAMP, MST mRNA level elevated and YAP protein and mRNA level were declined gradually, while pYAP protein level increased and TEAD1 mRNA level decreased. It demonstrated that SJAMP could prompt YAP phosphorylation through MST. The result of immunofluorescence analysis also indicated that after effected by SJAMP, there is a nuclear-to-cytoplasmic translocation of YAP. YAP assembles in cytoplasm and can’t go into the nucleus, which weakens the effect of YAP as a kind of cancer gene, inhibits the transcription of downstream genes.

We also found that after successfully knocked-down by YAP siRNA, the inhibition of proliferation of SJAMP to cancer cells was attenuated. Our previous study results indicated that SJAMP could decrease the expression of TEAD, while after knocked down the YAP by YAP siRNA, the expression of TEAD still decreased. That is to say that SJAMP had additional inhibition of TEAD expression even in the situation of knock-down of YAP. These results might mean that the SJAMP has other targets, not only YAP, to downregulate TEAD. Hippo-YAP pathway has a novel crosstalk with ERK and AKT pathway. These signaling pathways using CD44 as an upstream regulator function cooperatively to control downstream gene expression for cancer cell proliferation and cell cycle progression [19].

AKT can phosphorylate MST2 and LATS1 phosphorylates Raf-1, which were found to enable Raf-1 to suppress ERK and MST2 signaling [38]. AKT phosphorylates YAP lead to the nuclear export of YAP [39]. YAP also can induce ERK and AKT phosphorylation [17, 18]. Therefore, we proposed another hypothesis to link them together, which suggested that the signaling network consisting of ERK, AKT and Hippo-YAP participated in the inhibition effect of SJAMP to pancreatic cancer cell proliferation.

Caspase-9 is activated as an initial caspase, and then further induced the activation of the crack of Caspases and the cell apoptosis at last [40]. Survivin belongs to apoptosis inhibiting protein family. It receives apoptotic stimulating signal and inhibits cell apoptosis through caspase cascade [41], which roles in apoptosis is much alike with YAP [42]. It will be interesting to indicate whether YAP has tight relationship with Survivin in pancreatic cancer tissues. Our study demonstrated that SJAMP could obviously decrease Survivin and YAP mRNA level and raise the apoptosis factor caspase-9 mRNA level, which indicated that SJAMP could excite the mitochondrion apoptosis pathway and promote the pancreatic cancer cell apoptosis by inhibiting the expression of Survivin and prompting the level of Caspase-9.

In conclusion, we report that SJAMP, the marine medicine, has the effort of anti-tumor. It can inhibit SW1990 proliferation. Our data suggest the Hippo-YAP pathway has participate in these processes, implicating SJAMP potential therapy application to cancer therapy. We believe that SJAMP may also improve the effect of chemotherapy drug against pancreatic cancer. However, further clinical trials are needed to support our strategy for the treatment for pancreatic cancer.

## MATERIALS AND METHODS

### Cells culture

Pancreatic cancer cell lines SW1990 was cultured in RPMI 1640 (Invitrogen, Carlsbad, CA, USA) with 10% FBS in the condition of 37°C, 5%CO_2_. SJAMP was bought from the Medicine College of the Ocean University of China. When 90% confluent, the experimental groups were effected by SJAMP of 0.5, 1, 2, 4 and 8 mg/ml for 24 hs. Other experimental groups were affected by SJAMP of 8 mg/ml for 24 hs, 48 hs and 72 hs respectively. Cell population has been determined by MTT assay. The control group was incubated in the absence of SJAMP.

### Cell proliferation analysis

SW1990 proliferation was evaluated by MTT method (Sigma-Aldrich, USA). After affected by SJAMP of 0.5, 1, 2, 4, 8 mg/ml for 0, 24, 48, 72 hs, SW1990 were seeded into 96-well plates with 200 μl medium and 1 × 10^4^ cells per well. Changing fresh culture medium with MTT (5 mg/ml in PBS, 200 μl per well) at each time point, and then continue to incubate for another 4 h [43]. Following incubation, medium was discarded and dimethyl sulfoxide (DMSO 150 μl/well; Sigma-Aldrich) was added to each well for 10 mins. Absorbance at 490 nm is analyzed by an automated reader [43] (Bio-Rad, Hercules, CA, USA). To determine the effect of SJAMP to cell proliferation after treatment with YAP siRNA, the same procedures were performed to SW1990 with 8 mg/ml SJAMP for 24 h, 48 h, 72 h, after transfected with YAP siRNA.

### Patient information and tissue specimens

The 45 pairs of PDAC and adjacent non-tumor samples were used in this study which was diagnosed by pathological examination at the Affiliated Hospital of Qingdao University between 2011 and 2013. All individuals had be informed the purpose of our research and signed consent on the medical research use of their personal information and specimens according the authorization of the Institutional Research Ethics Committee. The individuals’ information and pathological characteristics were made a summary in Table 1. The clinical stages of pancreatic cancer were described using the tumor-node-metastases (TNM) cancer staging system announced by the International Union for Cancer Control (UICC) [44].

### Immunohistochemistry (IHC) staining

Human pancreatic cancer and adjacent non-tumor sections were incubated with rabbit polyclonal antibody to human YAP1 (1:100, Santa Cruz, USA) at 4°C overnight. The results of immunohistochemistry staining were scored by two experienced pathologists independently. Both the staining intensity and extent were considered. Staining intensity was scored from 0 to 3. Negative staining denotes 0; weak staining, light yellow, denotes 1; moderate staining denotes 2; and strong staining, deep yellow, denotes 3. [45] Percentage of positive tumor cells were counted to classify the extent of staining; (none, 0; 1–25%, 1; 26–50%, 2; 51–75%, 3; > 75%, 4). The immunohistochemistry staining results were defined into four levels considered both staining intensity and extent: negative (–), weakly positive (+), moderately positive (++), and strongly positive (+++), corresponding to 0, 1–4, 5–8, and 9–12.

### Small interfering RNA transfections

To Seed SW1990 into 6-well plates with a cell density of 2 × 10^5^ per well, and the siRNA was obtained from GenePharma (Shanghai, China). YAP siRNA and control sequences were as follows: YAP: 5′-GCAUC UUCGACAGUCUUCUTT3′ (sense), 5′-AGAAGACUGU CGAAGAUGCTT-3′ (anti-sense); control: 5′-UUCUCCG AACGUGUCACGUTT-3′ (sense), 5′-ACGUGACACGU UCGGAGAATT-3′ (anti-sense). According to the manufacturer’s instructions, transferring YAP siRNA into the SW1990 using Lipofectamine 2000 (Invitrogen, USA). Western blot was used to analyze the gene-silencing efficiency.

### Quantitative real-time PCR (qRT-PCR)

Samples from pancreatic cancer and adjacent non-tumor tissues and from different experiment cell groups were used to extract the total RNA by Trizol reagent (Invitrogen) Using 500 ng RNA to reverse transcript to cDNA using the RNA-to-cDNA kit (TaKaRa, USA). And then quantitative real-time PCR (qRT-PCR) reactions were carried out using the Power SYBR Green PCR Master Mix (TaKaRa, USA). The RT products were detected by PCR reaction which denatured for 30 seconds at 95°C, 5seconds for 95°C, 30s for 60°C by forty cycles in all. All the data were described using the comparative quantitative threshold cycle (ΔΔCt) method.

### Western blotting analysis

Equal quantities of denatured protein samples from different groups affected by SJAMP were extracted. After blocked with dry milk, polyvinylidene difluoride (PVDF) membranes were incubated with rabbit polyclonal antibody to human YAP, pYAP (1:1000; Santa Cruz, USA) antibodies at 4°C stay overnight. Using the Quantity One software to determine the band gray value and the average optical density ratio of YAP/GAPDH, pYAP/GAPDH to express the relative expression levels of protein.

The same procedures were performed after transfected with YAP siRNA and with SJAMP 4 mg/ml, 8 mg/ml for 24 hs and with 8 mg/ml SJAMP for 24 hs, 48 hs. Membranes were incubated with rabbit polyclonal antibody to human YAP, TEAD (1:1000; Santa Cruz, USA) antibodies at 4°C stay overnight. The ratios of YAP/GAPDH, TEAD/GAPDH were determined to express the relative expression levels of protein.

### Immunofluorescence

SW1990 cells were incubated with or without 8 mg/ml SJAMP for 48 hours and fixed for 15 min with 4% paraformaldehyde. And then the cells were permeabilized with 0.2% Triton X-100 for 7 min. After changed the medium, the cells were blocked with 5% bovine serum albumin (BSA) for 2 hours and incubated with YAP antibody at 4°C stay overnight. The next day, FITC were added in blocking solution for 1.5 hour at room temperature in the dark. Using 4′6′-diamidino-2- phenylindole (DAPI) to stain nuclei for 6 min. The cells were visualized by LSM510-Meta (Carl Zeiss AG, Germany).

### Statistic analysis

After gathering the data, all statistical analyses were implemented by Statistical Product and Service Solutions (SPSS) for windows 18.0 software (SPSS Inc., USA). The statistical significance analysis between two groups was estimated by the 2-tailed Student’s *t-test*. The significance of association between YAP immunohistochemistry expression in pancreatic cancer and histopathological variables were determined by Chi-square and Fisher exact test. The relationship between CA199 and YAP was evaluated using Pearson’s correlation test. To the all statistical analysis, *P-value* < 0.05 was considered to have significant difference.
